# The Effects of Press-Fit Technique Combined with Tranexamic Acid on Duration of Surgery and Intraoperative Blood Loss in Primary Total Hip Arthroplasty

**DOI:** 10.7759/cureus.23833

**Published:** 2022-04-05

**Authors:** Aziz Çataltepe, Kadir Öznam

**Affiliations:** 1 Department of Orthopedic Surgery and Traumatology, Medipol University, Istanbul, TUR

**Keywords:** intraoperative blood loss, operative time, tranexamic acid, press-fit technique, total hip arthroplasty

## Abstract

Background: Prolonged operative time and blood loss may affect the success rate in total hip arthroplasty (THA). The aim of the current study was to evaluate the effects of the press-fit (PF) technique without screws combined with tranexamic acid (TXA) on operative time and intraoperative blood loss in THA.

Methods: We retrospectively evaluated 114 hips treated with THA between March 2017 and January 2021 in this study. The patients were divided into three groups, including PF-TXA group, only PF group, and screw group. PF-TXA group received intravenous (IV) 1 g TXA 15 minutes before surgical incision, followed by a peri-articular 1 g/50 ml TXA. Only the PF group and screw group did not receive TXA. The primary outcome measures were operative time and intraoperative blood loss. Secondary outcomes included postoperative blood loss, hemoglobin and hematocrit levels, allogeneic blood transfusions, length of hospital stay, the Harris Hip Score (HHS), and thromboembolic complications.

Results: Operative time was lower in the PF-TXA group than that in the only PF and the screw group (p=0.0001). Intraoperative blood loss was significantly different in the PF-TXA group compared with the only PF and the screw group (423 ml, 516 ml, and 534 ml; respectively). The patients who received the PF technique combined with TXA had significantly less hospital stay length than the only PF group and the screw group (p=0.021).

Conclusion: The findings obtained in this study suggest that although only the PF technique can provide a shorter operative time compared to using screws, less blood loss may not be obtained using this technique in THA. PF technique combined with TXA significantly decreased operation time and intraoperative blood loss as well as the length of hospital stay following primary THA.

## Introduction

The initial stability of cementless implants is one of the main concerns for primary total hip arthroplasty (THA) with an uncemented technique [1]. The initial stability of the cementless acetabular component can be achieved with the press-fit (PF) technique without screws [2,3]. PF technique has some advantages, such as better surface contact at the bone-implant interface, decreased vascular or nerve damage, and shorter operative time in comparison with a screw technique [4,5]. PF technique without screws may shorten the duration of surgery, which can contribute to less bleeding in THA [5,6]. Shorter operative time decreases the risk of adverse events during THA, such as anemia, associated with blood loss, surgical site infection, extended hospital length of stay, and hospital readmission [7,8]. In addition, administering tranexamic acid (TXA) potentially can help the surgeon to perform the surgery faster [9].

A prolonged operation time in THA may lead to bleeding from bony and soft-tissue surfaces [8]. Significant intraoperative and postoperative blood loss may occur owing to hyper-fibrinolysis induced by surgical trauma after THA with screw or without screw [6,10]. Excessive blood loss may lead to complications and diminish the success of the surgery [11,12]. Substantial blood loss is associated with allogeneic blood transfusion, which has potential adverse effects on the body of patients, including the transmission of infectious diseases, immunologic reaction, transfusion-related acute lung injury, mistransfusion, and febrile reactions [12,13]. To reduce blood loss intraoperatively and postoperatively, many methods such as autologous blood transfusion, controlled hypotensive regional anesthesia, intraoperative blood salvage, shorter operative time, and TXA have been developed [9,14]. In the field of orthopedic surgery, TXA has been widely applied to total knee arthroplasty (TKA) and THA to reduce total blood loss and blood transfusion [15-17].

Operation time has been found to provide insufficient information regarding PF technique in THA. Furthermore, although TXA may decrease total blood loss and transfusion rates after THA, intraoperative blood loss remains controversial in the literature. Hence, we determined to combine the PF technique with IV and peri-articular TXA to minimize the operation time and blood loss. The primary purpose of this study was to evaluate the effects of the PF technique combined with TXA on operative time and intraoperative blood loss in THA.

## Materials and methods

After obtaining ethical approval from the local ethics committee of Medipol University (E-10840098-772.02-2915), a cohort of 113 consecutive patients who were performed on between March 2017 and January 2021 with unilateral THA for end-stage osteoarthritis were retrospectively assessed in the present study. Two patients diagnosed with rheumatoid arthritis and five patients diagnosed with Crowe type III or IV developmental dysplasia were excluded from this study. Two patients were lost to follow-up. Three patients died of unrelated causes to the surgery. Thus, 114 hips in 101 patients who met inclusion criteria were assessed. The patients over the age of eighteen years were divided into three groups. A total of 41 hips in the PF-TXA group received TXA, while a total of 37 hips in the only PF and a total of 36 hips in the screw group did not receive TXA. All patients stopped the anticoagulant and non-steroidal anti-inflammatory agents (NSAID) seven days before the surgery. Written informed consent was obtained from all patients who participated in this study.

The decision on anesthetic techniques, such as general or spinal with epidural anesthesia, was to rely on the anesthesiologists who did not participate in this study. All operations were performed with the same operative technique using a posterolateral approach. All hips were treated with the same cementless prosthesis (Trilogy cup, Versys Fiber Metal Taper stem; Zimmer, Warsaw, Indiana, USA). At the beginning of the surgery, the PF-TXA group received an IV loading dose of 1 g of TXA (Transamine, 250 mg/5 ml; Pharmacia, Teva, Turkey) in 100 ml of normal saline 15 minutes before surgical incision, followed by a peri-articular of 1 g TXA with 50 ml saline solution injected into the capsule and around the peri-articular soft tissue immediately after performing hip prosthesis and capsular closure and before skin closure. In all hips, we asked the anesthesiologist to reduce the blood pressure in a safe zone during the surgery as a strategy to minimize intraoperative blood loss until the wound closed. We calculated intraoperative blood loss by weighing sponges and suction drainage. In each group, the sole drain was placed into the hip joint and removed when the 24-hour volume of drainage was less than 50 ml after the surgery. After removing the drain, all of the patients began full weight-bearing walking using a walker postoperative day 1.

All of the patients were administered a standard course of an antithrombotic agent, which was low molecular-weight heparin (LMWH) (enoxaparin sodium, 40 mg) at eight hours postoperatively and continued three weeks after the surgery. Prophylactic antibiotic (cefazolin sodium, 1000 mg) was administered to all patients 30 minutes before the surgery and continued postoperatively over 24 hours.

Criteria of allogenic blood transfusion were a postoperative hemoglobin level of ≤ 8 g/dl, or a postoperative hemoglobin level between 8 and 10 g/dL with the clinical signs of hemodynamic instability, including light-headedness, presyncope, and palpitation or shortness of breath not owing to other causes. Pre-donation of autologous blood was not administered for any patients. We calculated total blood volume (TBV) using the Nadler method [18]. Estimated blood loss (EBL) was monitored using Gross’s formula, which considers the initial hematocrit before surgery, the minimum postoperative hematocrit level, and the average of the initial and minimum hematocrit levels [19]. Measured blood loss (MBL) was calculated as the sum of the intraoperative blood loss plus the total drain output. Operative time, intraoperative blood loss, EBL, length of hospital stay, thromboembolic complications, and demographic variables were assessed. The Harris Hip Score (HHS) was determined before operation and at the most recent follow-up.

Number Cruncher Statistical System (NCSS) 2007 Statistical Software (Utah, USA) program was used for statistical analysis. We expressed nominal data as frequencies or percentages and quantitative data as mean ± SD. The Shapiro-Wilk test was performed for testing the normality of study data. The one-way analysis of variance (ANOVA) test and the repeated one-way ANOVA test were used to detect differences between patients from each group for normal distributions. Groups were compared using the independent t-test for normally distributed continuous variables. The paired t-test was used to analyze preoperative and postoperative assessments of the HHS. The Chi-square test was used to analyze qualitative comparative parameters. We compared subgroups using Tukey’s multiple comparisons test. A p-value of ≤ 0.05 was considered statistically significant.

## Results

This retrospective comparative study involved 114 hips undergoing unilateral THA from 2017 to 2020 with a mean age of 58.2 years. All subject demographics, including age, gender, body mass index (BMI), American Society of Anesthesiologists (ASA) status, TBV, and preoperative hemoglobin levels, were similar among the three groups (p >0.05) (Table 1).

**Table 1:** Demographic characteristics of the patients. Values presented as mean (standard deviation) and p calculated by using ⁑the one-way analysis of variance (ANOVA) test, †the paired t-test, and +the Chi-square test. BMI: body mass index; ASA: American Society of Anesthesiologists; OA: osteoarthritis; AVN: avascular necrosis; DDH: developmental dysplasia of the hip; HR: heart rate; BP: blood pressure; HHS: Harris hip score.

Variable		PF-TXA Group n=41		Only PF Group n=37		Screw Group n=36		p-value
Mean age (year)		55.17±15.31		58.46±12.87		57.03±12.4		0.568⁑
Gender	Male	8	19.51%	12	32.43%	8	22.22%	0.385+
	Female	33	80.49%	25	67.57%	28	77.78%	
BMI		28.69±5.06		26.59±3.13		28.47±4.63		0.077⁑
ASA	1	22	53.66%	18	48.65%	17	47.22%	0.714+
	2	16	39.02%	14	37.84%	17	47.22%	
	3	3	7.32%	5	13.51%	2	5.56%	
Side (right/left)	Right	19	46.34%	22	59.46%	12	33.33%	0.082+
	Left	22	53.66%	15	40.54%	24	66.67%	
Diagnosis	Primary OA	28	68.29%	28	75.68%	25	69.44%	0.751+
	AVN	5	12.20%	2	5.41%	2	5.56%	
	Crowe I DDH	5	12.20%	3	8.11%	3	8.33%	
	Crowe II DDH	3	7.32%	4	10.81%	5	13.89%	
	Posttraumatic	0	0.00%	0	0.00%	1	2.78%	
Type of anesthesia	General	6	14.63%	9	24.32%	9	25.00%	0.451+
	Regional	35	85.37%	28	75.68%	27	75.00%	
BP		10.22±0.86		10.3±0.95		9.89±0.54		0.073⁑
HR		63.15±6.3		62.97±5.91		64.56±6.03		0.476⁑
Hospital stay (day)		3.15±0.36		3.30±0.46		3.44±0.56		0.021⁑
Total blood volume (ml)		4309.67±661.47		4346.43±586.76		4345.09±688.26		0.960⁑
Operation time (min)		73.49±8.42		81.03±7.46		92.33±10.07		0.0001⁑
HHS								
Preoperative		38.24±4.12		38.59±4.15		36.56±5.35		0.127⁑
Postoperative		88.27±5.9		90.27±5.5		88.72±5.93		0.288⁑
p-value†		0.0001		0.0001		0.0001		

The duration of the operation was lower in the PF-TXA group than the only PF group and the screw group (73.49±8.42 min, 81.03±7.46, and 92.33±10.07, respectively; p=0.0001) (Table 1). There was significantly less intraoperative blood loss found in the PF-TXA group compared to the only PF group and the screw group (257.93±67.25 ml, 395.14±79.34 ml, and 412.50±122.79 ml, respectively; p=0.0001). Furthermore, EBL was 990.01±166.97 ml in the PF-TXA group, significantly lower than 1362.13±312 ml in the only PF group and 1477.99±314.72 ml in the screw group (p=0.0001). Hemoglobin and hematocrit reduction were also significantly lower in the PF-TXA group (p=0.0001) (Table 2).

**Table 2:** Blood loss calculation, hemoglobin, hematocrit, and its reduction. Values presented as mean (standard deviation) and p calculated by using ⁑ the one-way analysis of variance (ANOVA) test and ‡the repeated one-way ANOVA test.

Variable	PF-TXA Group n=41	Only PF Group n=37	Screw Group n=36	p-value⁑
Intraoperative blood loss (ml)	257.93±67.25	395.14±79.34	412.50±122.79	0.0001
Drainage blood loss (ml)	312.32±101.61	459.41±132.61	510.28±138.74	0.0001
Estimated blood loss (ml)	990.01±166.97	1362.13±312	1477.99±314.72	0.0001
Hemoglobin (g/dl)				
Preoperative	13.00±1.19	13.14±1.18	13.01±1.17	0.849
Postoperative 1st day	11.37±1.16	11.31±1.23	11.20±1.06	0.810
Postoperative 2nd day	10.78±1.21	10.40±1.16	10.14±1.16	0.059
Postoperative 3rd day	10.32±1.09	9.62±1.14	9.16±1.11	0.0001
p‡	0.0001	0.0001	0.0001	
Hemoglobin reduction (g/dl)	2.1±0.43	3.51±0.54	3.82±0.69	0.0001
Hematocrit (%)				
Preoperative	39.27±3.57	39.65±3.31	39.10±3.38	0.777
Postoperative 1st day	34.43±3.33	34.23±3.47	33.81±3.11	0.714
Postoperative 2nd day	32.57±3.42	31.66±3.24	30.84±3.35	0.081
Postoperative 3rd day	31.17±3.18	29.07±3.18	27.82±3.13	0.0001
p-value‡	0.0001	0.0001	0.0001	
Hematocrit reduction (%)	8.19±1.23	10.66±1.45	11.4±1.89	0.0001

Whereas the only PF group had a shorter operation time compared to the screw group (p=0.0001), any significant differences regarding intraoperative blood loss, postoperative drainage volume, and total blood loss between the screw and PF group were observed (Table 3).

**Table 3:** Comparison of the three subgroups. P calculated by using Tukey’s multiple comparisons test.

Variable	Operation time (min)	Intraoperative blood loss (ml)	Drainage blood loss (ml)		Estimated blood loss (ml)
PF-TXA Group/Only PF Group	0.001	0.0001	0.0001	0.0001	
PF-TXA Group/Screw Group	0.0001	0.0001	0.0001	0.0001	
Only PF Group/Screw Group	0.0001	0.699	0.193	0.163	

Regarding the blood transfusion rate, six of 41 hips (14.63%) in the PF-TXA group received blood transfusions, whereas 13 of 36 hips (36.11%) in the PF group and 14 of 36 hips (38.89%) in the screw group received blood transfusions (Table 4).

**Table 4:** The correlation between blood transfusion and the length of hospital stay. Values presented as mean (standard deviation) and p calculated by using * the independent t-test.

Variable		N	Hospital stay (day)	p-value*
PF-TXA Group	Transfusion (-)	35	3.09±0.29	0.021
	Transfusion (+)	6	3.43±0.54	
Only PF Group	Transfusion (-)	24	3.04±0.21	0.0001
	Transfusion (+)	13	3.71±0.47	
Screw Group	Transfusion (-)	22	3.23±0.43	0.0001
	Transfusion (+)	14	3.79±0.58	

Patients who received the PF technique combined with TXA had significantly less length of hospital stay compared to the only PF group and the screw group (p=0.021). There was no significant difference among the three groups concerning the mean HHS score, which improved in all groups. In the PF-TXA group, one patient encountered pulmonary embolism (PE) treated with LMWH. One patient in the only PF group and two patients in the screw group experienced superficial wound infection, which was successfully treated with antibiotics. There was no neurovascular damage in all groups.

## Discussion

After evaluation of the findings, the main outcomes in the current study were a shortened operation time and reduced intraoperative blood loss in hips treated with PF and TXA. Operation time identified as a crucial independent factor may have an adverse effect on the clinical outcome of THA [20]. Småbrekke et al. suggested that the potential benefits of shorter operating times should be considered by orthopedic surgeons [21]. The duration of the surgical procedure can be modified to reduce complications after total joint arthroplasty [8]. Operation time which has been found insufficient information regarding PF technique for acetabular reconstruction is a changeable factor among orthopedic surgeons. PF technique without screws may shorten the duration of surgery, which can contribute to less bleeding [5,6]. Pepe et al. indicated that acetabular components with or without screws have consistent findings, but the use of screws increases the operation time significantly [6]. Similar outcomes were found in the current study in which only the PF group had a significantly shorter operative time compared to the screw group. Furthermore, Sandri et al. conducted a comparative retrospective study in which 40 patients received 1 g of IV-TXA before surgery, followed by 1 g of IV-TXA after surgery [9]. According to the findings obtained in this study, the mean operation time in the TXA group was significantly lower than in the control group. By contrast, Remérand et al. applied 15 mg/kg IV-TXA and found that although the operative time in the TXA group was shorter than the control group, there was no statistically significant difference between the two groups [22]. We applied the PF technique combined with TXA in the current study that showed that the mean operation time was significantly less compared with the only PF and the screw group. The operative time may be reduced using TXA and PF techniques owing to early hemostasis, better visibility of the operative field, and the advantage of not using screws which can save time. Moreover, prolonged operative time may be affected by a variety of factors such as an inexperienced surgeon, a generally slow-working surgeon, an inexperienced operating team, or a combination of these factors [21]. In the present study, the surgeon who had at least 18 years’ experience in hip arthroplasty surgery performed THA.

There are insufficient statistical data about intraoperative blood loss in THA fixed with PF fixation without screws in the literature. We obtained only one study conducted by Pepe et al. who did not find any significant difference regarding intraoperative blood loss and postoperative drainage volume and the total blood loss between the screw and PF group [6]. The same conclusion was drawn by the current study in which there was no significant difference concerning intraoperative and total blood loss between only the PF group and the screw group. In addition, although TXA may reduce total blood loss and transfusion rates after THA, intraoperative blood loss remains controversial in the literature. Claeys et al. and Johansson et al. performed a randomized controlled double-blinded trial examining the effects of IV-TXA on THA [23,24]. They reported that TXA was demonstrated a non-significant reduction in intraoperative blood loss and operation time compared to the control group. On the other hand, Ekbäck et al. showed that TXA significantly reduced intraoperative blood loss in the TXA group compared with the control group [25]. According to the current study, significant differences regarding shorter operation time and less intraoperative blood loss can be reached by combining the PF technique and TXA. To our knowledge, this is the first report that shows the effects of PF combined with TXA on operation time and intraoperative blood loss.

A prolonged operation time in THA may lead to bleeding from bony and soft-tissue surfaces [8]. Bohl et al. conducted a study in which there were 165,474 patients and found a strong relationship between operation time and anemia requiring blood transfusion [8]. The same conclusion was drawn by Salido et al. who showed that increased operative time had a higher risk of transfusion [26]. In this study, the patients who received blood transfusion had a longer operative time (96 min) than patients who did not receive a blood transfusion (87 min). Konig et al. applied topical TXA to reduce blood loss in 91 patients and compared it to 40 patients who did not receive TXA [27]. They showed that postoperative transfusions were reduced dramatically using TXA, dropping from 15% to 1% in THA. In the current study, we observed that the PF-TXA group had fewer blood transfusions than that in the only PF and the screw group.

The prolonged surgical duration may result in extended hospital length of stay [8,28]. Bohl et al. evaluated 165.474 knees and hips and found that an increase in operative time of 15 minutes was associated with an increase in the risk of anemia requiring transfusion by 9% and the risk of extended hospital length of stay by 9% [8]. Zeng et al. compared two groups (the TXA and the control group) and showed that intraoperative blood loss, total blood loss, and transfusion rates were significantly lower in the TXA group compared with the control group; however, length of stay after surgery did not show any significant difference [29]. In the present study, we found a statistically significant difference in the length of hospital stays in the PF-TXA group compared with the screw group, which had a mean longer operation time. The discrepancy in the length of hospital stay might be associated with patients who received blood transfusions, with a slightly slower postoperative recovery.

In the current study, the mean HHS was 88.27±5.9 in the PF-TXA group, 90.27±5.5 in the only PF group, and 88.72±5.93 in the screw group at the end of the study. Similar to the literature findings, the current study did not reveal any significant differences between the three groups. TXA did not increase the risk of thromboembolic complications, such as deep venous thrombosis (DVT) and PE, regardless of the administration route [30] However, in the current study, PE treated with LMWH occurred in one hip in the PF-TXA group. This hip was not administered any blood transfusion, but the patient has smoked two packages of cigarettes a day for 22 years. Moreover, when the open surgical site and the surgical instruments are exposed to longer operation time, bacteria from the operating room environment may pose severe problems for THA, including surgical site infection and wound dehiscence [8,28] One patient in the only PF group and two patients in the screw group experienced superficial wound infection, successfully treated with antibiotics.

We are aware that our data have some limitations. The main limitation of this study is its retrospective design. The number of included hips is relatively less, and this could result in the potential underpowering of the current study. Ultrasound examination could be applied at the follow-up to find asymptomatic DVT. All hips are performed by the same surgical and the same anesthetic team, which could reduce the potential surgical variability and perioperative blood loss.

## Conclusions

In conclusion, although only the PF technique can provide a shorter operative time compared to using screws, less blood loss may not be obtained using this technique in THA. The findings obtained in the present study suggested that the PF technique combined with TXA significantly decreased operation time and intraoperative blood loss as well as the length of hospital stay following primary THA.
